# Impact of Illness Perception on the Health-Related Quality of Life of Patients Receiving Dialysis: A Cross-Sectional Study

**DOI:** 10.7759/cureus.15705

**Published:** 2021-06-17

**Authors:** Abdulhameed A Alharbi, Yazeed A Alharbi, Ahmed S Alsobhi, Mohammad A Alharbi, Mariam A Alharbi, Adwaa A Aljohani, Alwaleed A Alharbi

**Affiliations:** 1 Joint Program of Preventive Medicine Post Graduate Studies, Ministry of Health, Medina, SAU; 2 Department of Internal Medicine, King Fahad Hospital, Jeddah, SAU; 3 Department of Internal Medicine, King Abdulaziz University Hospital, Jeddah, SAU; 4 Faculty of Medicine, King Abdulaziz University, Jeddah, SAU; 5 Health Affairs, Ministry of Health, Medina, SAU

**Keywords:** dialysis, end-stage renal disease, health-related quality of life, illness perception, saudi arabia

## Abstract

Background

End-stage renal disease (ESRD) is a major health problem worldwide that is increasing in incidence, prevalence, and cost. Both the disease itself and negative illness perceptions negatively affect patients' health-related quality of life (HRQoL), morbidity, and mortality. This study assessed the relationship between illness perception and HRQoL.

Methods

This cross-sectional study was conducted among 342 patients at five dialysis centers in Jeddah, Saudi Arabia. We used a self-administered questionnaire that containing demographic questions, the Revised Illness Perception Questionnaire, and the Short Form 36 Health Survey Questionnaire. The data were analyzed using t-tests, analyses of variance, Pearson's correlation coefficients, and multiple linear regression analyses.

Results

The mean (SD) age was 46.1 (16.5) years and the majority were men (53.8%). Except for treatment control, all domains of illness perception were significantly correlated with HRQoL; however, the correlations were positive only for personal control and illness coherence. Identity, disease timeline (acute/chronic), consequences, illness coherence, and emotional representations were independent predictors of HRQoL; together explaining 35% of the variance. Lower emotional response was the only domain of illness perception significantly associated with better HRQoL in both dialysis modalities across all dialysis centers.

Conclusion

There were clear effects of illness perception on HRQoL, with emotional representations being the strongest predictor. As such, emotional representations should be targeted in interventions.

## Introduction

End-stage renal disease (ESRD) is a major health problem worldwide that is increasing in incidence, prevalence, and cost [1-2]. ESRD is a debilitating, chronic condition that requires medical interventions, such as dialysis and some lifestyle, dietary, and fluid restrictions, for effective management [3-4]. For patients with ESRD, the blood-filtering function of the kidneys can be replaced by a medical procedure: dialysis [4-5].

The burden of illness among patients requiring dialysis can be aggravated by many factors such as treatment-related issues, limited activities, financial difficulties, and marital status stability. All these factors might increase emotional distress and affect the psychosocial wellbeing of the patients [5-6]. Patients with ESRD have a propensity to have a specific pattern of beliefs about their illness that can figure in their illness perception and the way they respond to it [4].

The illness perception of patients with a chronic disease is best described by the Self-regulatory Model (SRM) [5,7]. This model suggests that when a change in physical health takes place, individuals interpret their symptoms and create a hypothesis about their illness [8]. Accordingly, patients’ clinical outcomes and the ways of coping or controlling the symptoms is highly affected by the characteristics of an individual’s illness representation and beliefs about the illness and its treatment [8-11].

Patients' adaptive or maladaptive responses to an illness can be understood by identifying such beliefs and, consequently, improving adaptive functioning by altering such perceptions [5].

Health-related quality of life (HRQoL) is a key outcome indicator in research on patients with chronic illnesses, particularly in assessing the effects of different treatment modalities, including various forms of renal replacement therapy [12-13]. HRQoL generally serves as an indicator of patients’ wellbeing; however, it is also related to important clinical outcomes such as death [14].

The HRQoL of patients with chronic diseases, including ESRD, has been of particular interest [15]. ESRD, or indeed any chronic disease in combination with negative illness perceptions, can negatively affect patients' HRQoL, which can affect morbidity and mortality [5,11]. This has received some empirical support: poor HRQoL is strongly related to an increased risk of mortality among patients on dialysis [14,16].

Illness perception correlates differently with each of the eight domains of HRQoL: physical functioning, bodily pain, role-physical, role-emotional, emotional wellbeing, social functioning, energy/fatigue (vitality), and general health. Specifically, certain domains of illness perception are inversely correlated with HRQoL while others are positively correlated, as related below. Furthermore, the variance in HRQoL might be explained by only some of the domains of illness perception [5,17-18].

In a longitudinal study in the Netherlands, illness perception explained about 17% of the variance in the bodily pain and role-emotional domains of HRQoL and up to 51% of the variance in the mental health domain. Furthermore, each domain of HRQoL was explained by a different set of illness perception domains. Illness identity showed significant negative contributions to most domains of HRQoL. Consequences and emotional representations, however, showed strong negative associations with some of the domains [5]. Another three cross-sectional studies in Romania, Malaysia, and Iran, found that illness perception explained between 15% and 34% of the variance in the domains of HRQoL [17-18].

Over the last three decades, there has been a marked rise in the incidence and prevalence of ESRD in the kingdom of Saudi Arabia [19-20]. At the present rate, the number of patients with ESRD exceeds 20,000 [21]. Exploration of patients' illness perceptions and the effects of these perceptions on their HRQoL could help physicians tailor treatment plans to improve patients’ HRQoL. However, there is a dearth of studies on the illness perception of ESRD among patients receiving dialysis in Saudi Arabia or the effects of such perception on patients’ HRQoL. The aim of this study was to investigate the relationship between the HRQoL of dialysis patients and their illness perceptions.

## Materials and methods

Study setting and study population

This cross-sectional study was conducted at five dialysis centers in Jeddah city, Saudi Arabia. The inclusion criteria were having stable health (i.e., the ability to complete questionnaires), aged > 18 years, and having at least three or more months of dialysis. Patients who had a psychiatric disease were excluded.

Sampling

Proportionate stratification was used. First, we stratified participants by treatment modality, forming hemodialysis (HD) and peritoneal dialysis (PD) strata. Then, proportionate sampling was performed within each stratum. The calculated sample size was (n = 416). The equation used to calculate sample size was as follows: n = ⱬ² p(1-p)/δ2, where n = sample size, ⱬ = ⱬ score (i.e., 1.96 for a 95% confidence level), p = proportion, and δ = confidence interval (which reflects precision). The assumed proportion was set at 0.5 to obtain the maximal sample size while the worst acceptable value (δ) was set at 0.05. This resulted in a sample size for an infinite population of 384. To adjust the sample for a finite population, the sample size for each stratum was calculated using the following equation: nᵢ = n˳(1 + (n˳-1)/N), where nᵢ = stratum sample, n˳= calculated sample size for an infinite population, and N = stratum population. At each dialysis center, patients at each dialysis session were sorted alphabetically in tables to ensure homogeneity. Then, using simple random sampling, participants were selected according to the sample size of each stratum at each dialysis center.

Study instruments

Together with socio-demographic data, two self-administered questionnaires were provided to participants: the Revised Illness Perception Questionnaire (IPQ-R) [22] and the Short Form 36 Health Survey Questionnaire (SF-36) [23], which were integrated into a single questionnaire package in Arabic. The part of the questionnaire on demographic information was preceded by an introduction requesting for patients to fill in the questionnaire during their dialysis sessions, without compensation, and to include their participation agreement and personal data. The socio-demographic data r included age, sex, educational level, marital status, and employment status. The second part of the questionnaire contained the well-validated IPQ-R. Currently, the IPQ-R is the most common instrument used among patients with chronic diseases for the assessment of illness perceptions [24]. It is an 84-item instrument that assesses the different domains of illness perception. It has three parts and nine subscales [25].

The first part includes one subscale, identity, that is used to assess the symptoms associated with patients' condition. Patients were asked about 14 symptoms and if they thought that symptoms were related to their illness. The number of yes-rated items forms the score of the identity subscale [25-26].

The second part includes 38 items, which are divided into seven subscales: timeline acute/chronic, timeline cyclical, consequences, personal control, treatment control, illness coherence, and emotional representations. Each item was rated on a five-point Likert scale, ranging from 1 (“strongly disagree”) to 5 (“strongly agree”). The overall score was obtained by the sum of the items of each subscale. The scores of 13 items out of 38, were reversed [25].

High scores on the consequences, emotional representations, and timeline subscales indicate a more negative perception of the illness [27]. That means that patients view the illness as chronic and cyclical in nature and believe their illness to be serious outcomes and they have strong emotional reactions to it. Each item was rated on a five-point Likert scale, ranging from 1 (“strongly disagree”) to 5 (“strongly agree”). More positive perceptions about the controllability and understanding of the illness is indicated by high scores on the treatment control, personal control, and coherence subscales [27].

The third section includes one subscale, the causal subscale, which is concerned with 18 possible causes that might be attributed by patients to their current illness. The mean of each item is computed, and a higher mean indicated a more common cause [27].

Forward-backward translation by two experts was used to translate the IPQ-R into the Arabic language.

The final part of the study questionnaire was the Arabic version of the SF-36. This is a widely used validated instrument for assessing HRQoL [23]. The SF-36 assesses eight domains of HRQoL: physical functioning, bodily pain, role-physical, role-emotional, emotional wellbeing, social functioning, energy/fatigue (vitality), and general health. Of the 36 items, one item assesses the change in general health over a one-year period [28]. The Cronbach's alphas for the Arabic RAND-36 in multiple subgroups exceeded 0.70 for every scale except general health (Cronbach's alpha = 0.59) [28].

The SF-36 is scored using a two-step process. First, all items are scored according to a given key. The Likert score of each item is then transformed to a range from 0 to 100. In step two, items in the same subscale are average together to create subscale scores. Items that are left blank are not considered when calculating the subscale scores. Therefore, each subscale score represents the mean of possible scores achieved in that subscale. The Arabic validated version of the SF-36 was used in this study.

Procedure

The study purpose and instructions about filling the questionnaire were explained to the participants. For illiterate participants, the data was collected through a face-to-face interview.

Ethical considerations

Permission for translating to Arabic and using the validated questionnaires was obtained from the original developer of the IPQ-R, Professor Rona Moss-Morris [22]. RAND Corporation, based in California, USA, allows anyone to use the SF-36, and it has been translated into a number of languages, including Arabic [29].

Ethical approval was obtained from each center. Participants were informed that participation was optional and that they could refrain from answering any question or the whole questionnaire if they so desired. Furthermore, participants were informed that information are confidential and would not be disclosed except for research purposes.

Statistical analyses

IBM SPSS software Version 20 (IBM Corp., Armonk, NY) was used for data analysis. Mean, SD, frequencies, and percentages were obtained in descriptive analysis. Pearson's correlations were calculated between the various subscales of the IPQ-R and SF-36. A backward multiple linear regression analysis was conducted (unlike forward stepwise analysis, it begins with the full least squares model containing all predictors, and then consecutively removes the least useful predictors) to determine the independent predictors of HRQoL. Variables that were not correlated with HRQoL in the correlational analysis were excluded as predictors from the regression analysis. Significance was set at P ≤ .05. The basic assumptions for performing regression analyses (i.e., assumptions of normal distribution, a linear relationship between the independent and dependent variables, reliability, and homoscedasticity) were met [30].

## Results

Participants’ characteristics

The overall response rate was 82.2%. The majority were men (53.8%), single (56.1%), and unemployed (74.2%). The mean (SD) age was 46.2 (16.4) years (Table 1).

**Table 1:** Personal and professional characteristics of the participants

Characteristic	n	%
Sex		
Male	184	53.8
Female	158	46.2
Marital status		
Married	96	28.3
Single	190	56.1
Divorced	19	5.6
Widowed	34	10
Education level		
Illiterate	76	22.5
Just read and write	21	6.2
Primary school	43	12.7
Intermediate school	46	13.6
High school	86	25.4
Bachelor's degree or higher	66	19.5
Age (Mean ± SD)	46.1 ± 16.5 years	
Occupation		
Unemployed	219	74.2
Employed	76	25.8

Illness perceptions scores

Regarding the IPQ-R score, the mean (SD) timeline cyclical was 3.42 (0.72), mean (SD) consequences was 3.36 (0.79), and mean (SD) illness coherence was 3.35 (0.80). This means that participants perceived their symptoms as changing over time, that their disease had an impact on their life, and that they perceive their conditions well. The mean (SD) personal control subscale was 3.24 (0.52), mean (SD) treatment control subscale was 3.32(0.49), and mean (SD) emotional representation subscale was 3.28 (1.0). The mean (SD) timeline acute/chronic score was 3.03 (0.79), which indicates that perceived their illness as chronic (Table 2).

**Table 2:** Mean scores and standard deviations for the Revised Illness Perception Questionnaire (IPQ-R)

Subscale	Items mean (SD)	Score range	Mean (SD)	%
Identity		0 – 14	6.90 (3.29)	49.3%
Timeline acute/chronic	3.03 (0.79)	6 – 30	18.2 (4.77)	60.7%
Timeline cyclical	3.42 (0.72)	4 – 20	13.7 (2.87)	68.5%
Consequences	3.36 (0.79)	6 – 30	20.2 (4.71)	67.3%
Personal control	3.24 (0.52)	6 – 30	19.5 (3.14)	64.9%
Treatment control	3.32 (0.49)	5 – 25	16.6 (2.43)	66.5%
Illness coherence	3.35 (0.80)	5 – 25	16.7 (3.99)	67.0%
Emotional representation	3.28 (1.00)	6 – 30	19.7 (6.02)	65.6%

Scores of HRQoL

As shown in Table 3, patients receiving dialysis showed lower scores on the SF-36 subscales of role-physical (32.5%) and role-emotional (39.3%) in comparison to other subscales. In contrast, the best scores were for emotional wellbeing (59.9%) and social functioning (57.4%).

**Table 3:** Mean scores and standard deviations for the SF-36

Subscale	Mean	SD
Physical functioning	49.3	28.2
Role-physical	32.5	38.4
Role-emotional	39.3	42.9
Energy/fatigue	43.7	20.0
Emotional wellbeing	59.9	21.3
Social functioning	57.4	26.6
Pain	55.7	28.8
General health	47.9	16.1
Health change	55.8	30.4
Total (HRQoL)	48.2	18.8
Subscale	Mean	SD
Physical functioning	49.3	28.2
Role-physical	32.5	38.4
Role-emotional	39.3	42.9
Energy/fatigue	43.7	20.0
Emotional wellbeing	59.9	21.3
Social functioning	57.4	26.6
Pain	55.7	28.8
General health	47.9	16.1
Health change	55.8	30.4
Total (HRQoL)	48.2	18.8

Impact of illness perceptions on HRQoL

For all patients receiving dialysis, significant correlations were found between HRQoL and all subscales of the IPQ-R, except for treatment control. However, the correlations of HRQoL with identity, timeline acute/chronic, timeline cyclical, consequences, and emotional representations were negative. In other words, low HRQoL in patients receiving dialysis was associated with a stronger belief in the chronicity and cyclical nature of the illness, that the illness has serious consequences, and that the illness provoked a stronger emotional response.

In contrast, HRQoL was positively correlated with personal control and illness coherence. Thus, stronger beliefs in having a role in managing the illness and a better understanding of the illness were associated with better HRQoL (Table 4).

**Table 4:** Pearson’s correlations between the IPQ-R subscales and SF-36 total score (HRQoL)

Subscale	HRQoL	
	R	P (2-tailed)
Identity	-0.303	< .001
Timeline acute/chronic	-0.217	< .001
Timeline cyclical	-0.296	< .001
Consequences	-0.440	< .001
Personal control	0.191	< .001
Treatment control items	0.063	0.248
Illness coherence items	0.320	< .001
Emotional representations	-0.503	< .001

Predictors of HRQoL

Table 5 shows that of the nine independent predictors of IPQ-R subscales, only five contributed significantly to the variance in HRQoL: identity, timeline acute/chronic, consequences, illness coherence, and emotional representations. Illness coherence was a positive predictor while the others were negative predictors. These five factors explained 35% of the variance in HRQoL.

**Table 5:** Regression analysis of IPQ-R subscales predicting SF-36 total score (HRQoL)

Independent variable	β	SE	Beta	P	95% CI for β
(Constant)	89.68	7.2		< .001	75.48, 103.88
Identity	-.907	.26	-.158	.001	-1.427, -.387
Timeline acute/chronic	-.459	.17	-.116	.011	-.811, -.108
Consequences	-.739	.21	-.185	.001	-1.155, -.324
Illness coherence	.477	.23	.101	.041	.019, .934
Emotional representations	-1.017	.16	-.324	< .001	-1.346, -.687

In addition, backward regression (Table 6) of the demographic factors (sex, age in years, marital status, education level, and occupation) and dialysis characteristics (duration, modality, and center) showed that age, marital status, and dialysis modality were additional independent predictors of the variance in HRQoL. However, together, they explained just 7% of the variance in HRQoL.

**Table 6:** Regression analysis of demographic and dialysis factors predicting SF-36 total score (HRQoL)

Independent variable	β	SE	Beta	P	95% CI for β
(Constant)	54.4	4.6		< .001	45.399, 63.506
Dialysis modality	5.3	2.5	.118	.038	.295, 10.385
Age in years	-4.8	1.4	-.186	.001	-7.712, -1.919
Marital status	-2.7	1.0	-.143	.012	-4.861, -.592

## Discussion

According to participants' scores on the IPQ-R, participants attributed a low proportion of their symptoms to their illness. They experienced ESRD as a chronic illness that had a negative impact on their lives. Participants were unstable emotionally, though they felt that they could control their illness through their own behavior and treatment. Participants in this study showed an average IPQ-R score, stronger emotional representations, and more symptom variation over time in comparison to patients with other chronic diseases [26,31-35]. Patients with ESRD may experience stronger emotional upset than patients with other illnesses, owing to treatment-related symptoms and the presence of the arterio-venous fistula, which keeps reminding the patients about their need for dialysis [36].

Compared to the general population [28], participants displayed low scores on all subscales of the SF-36. Essentially, this is owing to the burden of the symptoms of their illness and dialysis [37-38]. More importantly, compared to other chronic illnesses, such as chronic gastritis [39] or diabetes mellitus [40], participants reported lower HRQoL in general and greater defects in physical functioning. This might be because patients with ESRD require dialysis on a near-daily basis, with each session lasting for around four or five hours and frequently accompanied by unpleasant symptoms. In addition, potentially treatable symptoms of ESRD are often undertreated [41]. Patients on regular dialysis have reported around nine symptoms (e.g., fatigue, muscle weakness, and sleep difficulty) that resulted in impaired HRQoL [42]. According to another study, the symptom burden of ESRD is similar to that of terminal cancer [43].

The major aim of the current study was to investigate the impact of illness perception on HRQoL. Except for treatment control, all domains of illness perception showed significant correlations with HRQoL. However, the correlations were negative for identity, timeline acute/chronic, timeline cyclical, consequences, and emotional representations. In contrast, personal control and illness coherence showed positive correlations with HRQoL. Accordingly, better HRQoL is associated with a weaker belief in the chronicity and cyclical nature of the illness, perceiving the illness to have serious consequences, and weaker emotional responses. In addition, better HRQoL is associated with a stronger belief in having a role in illness improvement and a better understanding of the illness. This result is consistent with one Malaysian study concerning patients receiving HD [11] and partly with an Iranian study [11]. The latter study showed the same negative correlations but no positive correlations among 120 patients receiving HD.

Five domains of illness perception were independent predictors of the variance in HRQoL: identity, timeline acute/chronic, consequences, illness coherence, and emotional representations. Illness coherence was a positive predictor while the others were negative predictors; together, 35% of the variance in HRQoL was explained by these five factors. Despite variations between previous studies and this study regarding the names and number of illness perception domains predicting the variance in HRQoL, research is relatively consistent concerning the magnitude of the effects [5,17-18]. The variations in predictors between studies were likely because most studies dealt solely with patients receiving HD. Furthermore, they typically divided HRQoL into the physical component summary and mental component summary subscales; since there is no agreed-upon reference for those components, we instead relied on the approved scoring key.

Emotional representation was the only domain of illness perception that was significantly associated with better HRQoL in both dialysis modalities and in all dialysis centers. Consequently, it can be considered the best predictor of HRQoL, which accords with the findings of previous studies [5,17-18]. Thus, how patients react to their disease or treatment is reflected indirectly in their HRQoL. This follows the SRM, which proposes that patients attempt to reduce their illness in ways that are consistent with their own emotions [9,44-45].

Limitations

The cross-sectional design of the study was its major limitation, which precluded an assessment of the temporal priority or causality of the observed correlations. Clinical parameters (e.g., hemoglobin level and dialysis adequacy) were not included in the current study.

## Conclusions

There was a clear effect of illness perception on HRQoL, with emotional representations being the strongest predictor. Age, marital status, and dialysis modality were additional independent predictors of HRQoL. There is a need for an intervention program to support those patients to improve their quality of life. Further, before beginning an intervention program for patients receiving dialysis, it might be better to explore the effect of illness perception on dialysis treatment. From there, an intervention program could be constructed to target each domain of illness perception in a manner best suited to improving the HRQoL of each patient. Specifically, the emotional response of patients receiving dialysis is the most important domain of illness perception; therefore, it should receive considerable attention in interventions for improving HRQoL. Future studies are required to examine clinical parameters in more detail. A longitudinal design would provide greater detail about the relationship between illness perception and HRQoL.
